# Impact of continuous Kangaroo Mother Care initiated immediately after birth (iKMC) on survival of newborns with birth weight between 1.0 to < 1.8 kg: study protocol for a randomized controlled trial

**DOI:** 10.1186/s13063-020-4101-1

**Published:** 2020-03-19

**Authors:** WHO Immediate KMC Study Group, Ebunoluwa Aderonke Adejuyigbe, Pratima Anand, Daniel Ansong, Chineme Henry Anyabolu, Sugandha Arya, Evelyne Assenga, Ibraheem Awowole, Monika Bahl, Rajiv Bahl, Jill Bergman, Nils Bergman, Adwoa Boakye-Yiadom, Rahul Chauhan, Harish Chellani, Nidhi Chopra, Rupali Dewan, Queen Dube, Luis Gadama, Harsh Vardhan Jaiswal, Kondwani Kawaza, Bankole Peter Kuti, Oluwafemi Kuti, Roderick Larsen-Reindorf, Agnes Linnér, Alexander Manu, Augustine Massawe, Nicole Minckas, Pratima Mittal, Ausbert Msusa, Helga Naburi, Sam Newton, Matilda Ngarina, Ayodele Olanrewaju Oladele, Gyikua Phlange-Rhule, Cynthia Pillegi-Castro, Nisha Rani, P. N. Suman Rao, Siren Rettedal, Isha Saini, Vincent Samuel, Richa Singhal, Jyotsana Suri, Nitya Wadhwa, Björn Westrup, Naana Wireko-Brobby, Sachiyo Yoshida

**Affiliations:** World Health Organization, 20, Avenue Appia, CH1211 Geneva, Switzerland

**Keywords:** Immediate Kangaroo Mother Care (iKMC), Low-birth-weight babies, Mortality, Skin-to-skin contact, Breastfeeding, Mother-neonatal intensive care unit (M-NICU)

## Abstract

**Background:**

Globally, about 15% of newborns are born with a low birth weight (LBW) as a result of preterm birth or intrauterine growth restriction or both. Up to 70% of neonatal deaths occur in this group within the first 3 days after birth. Kangaroo Mother Care (KMC) applied after stabilization of the infant has been shown to reduce mortality by 40% among hospitalized infants with a birth weight of less than 2.0 kg. In these studies, infants were randomly assigned and KMC was initiated after about 3 days of age, when the majority of neonatal deaths would have already occurred. The aim of this trial is to evaluate the safety and efficacy of continuous KMC initiated as soon as possible after birth compared with the current recommendation of initiating continuous KMC after stabilization in neonates with a birth weight between 1.0 and less than 1.8 kg.

**Methods:**

This randomized controlled trial is being conducted in tertiary-care hospitals in five low- to middle-income countries (LMICs) in South Asia and sub-Saharan Africa. All pregnant women admitted to these hospitals for childbirth are pre-screened. After delivery, all neonates with a birth weight between 1.0 and less than 1.8 kg are screened for enrollment. Eligible infants are randomly assigned to intervention and control groups. The intervention consists of continuous skin-to-skin contact initiated as soon as possible after birth, promotion and support for early exclusive breastfeeding, and provision of health care for mother and baby with as little separation as possible. This efficacy trial will primarily evaluate the impact of KMC started immediately after birth on neonatal death (between enrollment and 72 h of age and deaths between enrollment and 28 days of age) and other key outcomes.

**Discussion:**

This is the first large multi-country trial studying immediate KMC in LMICs. Implementation of this intervention has already resulted in an important enhancement of the paradigm shift in LMIC settings in which mothers are not separated from their baby in neonatal intensive care units (NICUs). The findings of this trial will have future global implications not only on how the LBW newborns are cared for immediately after birth but also for the dissemination of designing NICUs in accordance with the mother-neonatal intensive care unit (M-NICU) model.

**Trial registration:**

Clinical Trials Registry - India (CTRI): CTRI/2018/08/01536 (retrospectively registered); Australian New Zealand Clinical Trials Registry (ANZCTR): ACTRN12618001880235 (retrospectively registered).

## 1. Background

Globally, about 15% of infants are low birth weight (LBW) as a result of preterm birth or intrauterine growth retardation or both, and 60 to 80% of deaths in the neonatal period occur in these LBW infants [1–3]. The majority of neonatal deaths occur within the first 3 days of life. The causes of death among LBW infants include respiratory and brain complications, hypothermia, hypoglycemia, and infection [4, 5], which occur as a result of the immaturity of their lungs, brain, gastrointestinal tract, and skin.

Several interventions have been proven to be effective for improving survival of LBW infants, such as antenatal corticosteroids, breastfeeding, hygiene, case management of suspected infections, and hospital care of small babies, including Kangaroo Mother Care (KMC) [6]. The World Health Organization (WHO) defines KMC as the care of preterm and LBW infants where the mother keeps the baby in skin-to-skin contact (SSC) on her chest continuously until the baby no longer wants to stay in that position and she exclusively breastfeeds the baby [7]. Preterm infants have fundamental challenges to maintain thermoregulation and become cold very rapidly after birth and to a great extent this can be prevented by SSC. Evidence shows that KMC reduces mortality, possibly by helping maintenance of better thermoregulation, facilitating the earlier initiation of breastfeeding, reducing the risk of nosocomial infection, reducing the risk of apneic attacks, and promoting bonding of the mother–infant dyad [8–19].

The WHO publication *Kangaroo Mother Care: A Practical Guide* recommends initiation of short KMC sessions when the baby starts to recover, even if the baby still requires medical treatment such as intravenous fluids or oxygen [7]. It also recommends that continuous KMC should be initiated only after the baby is stable, meaning that the baby must be breathing spontaneously without additional oxygen. For the routine care of newborns weighing 2.0 kg or less at birth, the “WHO recommendations on interventions to improve preterm birth outcomes” recommend KMC, which should be initiated in health-care facilities as soon as the newborns are clinically stable [20]. These babies should be provided with as close to continuous KMC as possible. Currently, there is no recommendation for KMC for unstable neonates weighing less than 2.0 kg at birth.

A recently updated Cochrane review reported 40% lower mortality in infants with a birth weight of less than 2.0 kg who were given KMC as compared with those who were given standard (conventional) care in hospitals at 40–41 weeks’ postmenstrual age (3.2% versus 5.3%; risk ratio (RR) 0.60, 95% confidence interval (CI) 0.39 to 0.92; eight trials, 1736 infants) [21]. This review also showed a 65% relative reduction in the occurrence of nosocomial infections or sepsis at 40–41 weeks’ gestational age (RR 0.35, 95% CI 0.22 to 0.54; five trials, 1239 infants), a 30% improvement in exclusive breastfeeding at the end of neonatal period (RR 1.30, 95% CI 1.12 to 1.49: six trials, 711 infants), a shorter duration of hospital stay, and a higher prevalence, duration, and exclusivity of breast feeding [22]. In almost all studies included in the Cochrane review, KMC was initiated after the baby was clinically stable. For most studies, the median age at initiation of KMC was 3.2 to 24.5 days. Only in one study was KMC initiated before stabilization at a median age of 10 h [23]. This implies that over two thirds of deaths among preterm babies would have occurred by the time these infants became stable enough to be provided KMC [24].

KMC cannot influence deaths that happen before its initiation. Thus, the 40% mortality impact of KMC in enrolled infants would translate in practice to only about 13% impact on mortality in all babies with a birth weight of less than 2.0 kg. Only two small randomized controlled trials (RCTs) have evaluated the feasibility, safety, and effect on stabilization of initiating KMC immediately after birth. In the RCT from South Africa [25], SSC from birth was associated with 100% stability scores in the fifth to sixth hour of life as compared with 46% in the conventional care group in newborns weighing 1.2–2.2 kg. A similar RCT from Vietnam in neonates weighing 1.5–2.5 kg reported significantly better transition to extra-uterine life (*P* <0.02) in the immediate SSC group. The neonates in the intervention group had significantly lower need for respiratory support, intravenous fluids, and antibiotic use during their hospital stay [26].

The impact of the KMC intervention could have been much larger had it been initiated immediately after birth. However, this has not been evaluated in LBW infants. Therefore, we aim to evaluate the safety and efficacy of continuous KMC initiated immediately after birth for neonates with a birth weight of 1.0 to less than 1.8 kg compared with initiating KMC after stabilization in improving survival. In this study, our main hypothesis is that those very small babies who are provided continuous KMC initiated immediately after birth will experience a reduced risk of death compared with a similar group in whom KMC is initiated only after stabilization.

## 2. Methods

### 2.1. Objectives and trial design

This is a multi-country, multi-center, non-blinded RCT to measure the effect of continuous KMC initiated immediately after birth on post-randomization mortality during the first 72 h of life and during the neonatal period, compared with continuous KMC initiated after stabilization, in infants with a birth weight of at least 1.0 and less than 1.8 kg born in hospitals in low- to middle-income countries (LMICs). The secondary objectives are to determine the effect of the intervention on time to clinical stabilization, hypothermia, time taken to reach full breastfeeding, hypoglycemia, clinically suspected sepsis, time to hospital discharge, exclusive breastfeeding at the end of the neonatal period, maternal satisfaction with health care in the hospital, and maternal depression at the end of the neonatal period. The nature of the intervention makes blinding not possible.

### 2.2. Study setting

The study is being conducted in five tertiary-level hospitals in low-resource countries of Asia and sub-Saharan Africa, including Ghana, India, Malawi, Nigeria, and Tanzania. These hospitals were selected because they serve as referral centers capable of providing care to women at risk of delivering small babies and have a high proportion of LBW infants. In all of these hospitals, babies with a birth weight of less than 1.8 kg are routinely separated from the mother just after birth and provided care in neonatal intensive care units (NICUs). Care provided in NICUs includes warmth, breast-milk feeding as per availability, and (if required) intravenous fluids, parenteral antibiotics, oxygen, and continuous positive airway pressure (CPAP). There is limited or no access to more sophisticated interventions such as surfactant therapy and mechanical ventilation in most hospitals. Routine KMC is practiced in all of these hospitals and is initiated after achieving stabilization, usually after 3–7 days of age. The quality of care in the participating hospitals has been upgraded though the training of staff on the WHO minimum package of care for small babies. Identical weighing scales, mobile and fixed monitoring equipment, and CPAP equipment have also been provided to all sites, and training in their use was provided by a team of neonatologists.

### 2.3. Study population

All infants born alive in the participating hospitals with a birth weight from 1.0 to less than 1.8 kg, regardless of their gestational age, are eligible for participation in this trial with their mothers. The mother–infant pair is eligible even if the infants are twins or are born by caesarean section or if the mother experiences some complications during labor and delivery that are expected to be resolved within 3 days.

A mother–infant pair is not eligible if any of the following is present: (1) the mother is younger than 15 years of age, (2) the mother (or her guardian if the mother is 15–17 years old) is unable or unwilling to provide consent, (3) the mother is unlikely to be able to provide KMC within the first 3 days after birth (e.g., she has eclampsia or shock or has undergone major surgery), (4) the baby is unable to breathe spontaneously within 1 h of birth, (5) triplets or more, (6) the baby has a congenital malformation that interferes with the intervention or the intervention interferes with the required care for the congenital malformation, (7) the place of residence is not a part of the defined study area (the study area has been defined to make 28-day follow-up home visit feasible), or (8) if for any reason the mother–infant pair cannot be enrolled within 2 h of the birth of the infant.

### 2.4. Sample size calculation

Preliminary unpublished data obtained from the admission and discharge registers of the five selected hospitals show that neonatal mortality among infants with birth weights from 1.0 to less than 1.8 kg was about 32% in 2015. After improved implementation of the WHO-recommended minimum package of care for LBW infants in these hospitals, we expect mortality in the control group to fall by about one third (to be about 21%) during the study period. We calculated the sample size hypothesizing a 20% reduction in mortality (mortality in the control group: 21%; expected mortality in the intervention group: 16.8%) with a power of 90% and a significance level of 5%, allowing a maximum loss to follow-up of 10%. The sample size for comparison of two proportions is 2080 per group; thus, 4200 neonates are required to be enrolled.

### 2.5. The intervention

Formative research was conducted in each site to identify barriers to delivering and accepting the intervention and to develop and test solutions to overcome these. In this study, KMC is defined as continuous SSC with the mother or her surrogate aiming for at least 20 h per day, support for exclusive breastfeeding, and required medical care without separation from the mother as much as possible. The surrogate is a female relative or friend identified by the mother to provide SSC when she is unable to do so. Therefore, the intervention consists of three components:

1. *Promotion and support for continuous SSC initiated as soon as possible after birth*: Continuous SSC is initiated immediately after randomization, as soon as feasible after birth, aiming for at least 20 h a day. This is initiated by the mother or the surrogate within the delivery room or the operation theatre or on admission to the NICU and continued during transfer to the NICU and during the stay in the NICU. Mother and infant are kept in the neonatal unit until the infant meets predefined stability criteria. An infant is considered stable when he or she is breathing spontaneously with no oxygen or CPAP support at 40–60 breaths per minute, maintaining oxygen saturation on room air more than 90%, does not have apnea, has a heart rate of 80 to fewer than 180 beats per minute, an axillary temperature of 36.0 to 37.4 °C and does not need intravenous fluids. After stabilization, the mother–infant dyad is shifted from the NICU to the KMC ward, where continuous KMC is provided until discharge from the hospital.
2. *Health care for mother and infant provided without separation*: The mother and infant are provided health care without separation as much as possible. Mothers are provided a place to sleep, food and health care by obstetric staff while in the NICU. If a mother has any complication for which she needs to be transferred to the obstetric ward or intensive care unit, SSC is continued with a surrogate until the mother becomes available. If the infant requires a procedure or treatment that is not possible in SSC, the infant is shifted to a cot or radiant warmer. SSC is temporarily interrupted for the period of the procedure or treatment and recommenced as soon as possible after that.
3. *Promotion and support for early and exclusive breastfeeding*: Mothers are encouraged and supported to put the infant to the breast when they are in the NICU. Even if the infant is unable to feed from the breast, putting the infant to the breast provides the infant the opportunity to learn how to attach and suckle. When possible, early expression and feeding of colostrum are carried out. A breastfeeding counsellor is available at all sites to help the mothers solve breastfeeding problems they face.

### 2.6. Comparator

Neonates randomly assigned to the control group receive conventional care, and in accordance with the routine at the sites, the mother and infant are separated until the baby is clinically stable. Except for the time of initiation of KMC, all other medical care is the same for intervention and comparison groups. When feeding can be started on the basis of the clinical condition of the baby, expressed breast milk is given using a feeding tube or cup and direct breastfeeding is started when the baby is ready. Short sessions of KMC are started for neonates randomly assigned to the control group when the baby is considered to be recovering (off CPAP, oxygen requirement of less than 30% and tolerating partial enteral feeds) and is at least 24 h old. The mother comes to the NICU to provide these brief sessions of KMC a few times a day during the time allocated for infant feeding. Continuous KMC is initiated for an infant randomly assigned to the comparison group when the infant meets stability criteria for at least a continuous period of 24 h and can be transferred to the KMC ward. As in the intervention group, mother and infant are kept in the neonatal unit until the infant meets the stability criteria. A minimum package of care for the neonates and the mothers is provided as a co-intervention for both the intervention and the control groups.

### 2.7. Primary and secondary outcomes

The primary outcomes of this study are mortality between enrollment and 72 h and mortality between enrollment and 28 days of age. Secondary outcomes are presented in Table 1. All outcomes are measured by using identical methods in the intervention and control groups by an independent outcome measurement team, which is not involved in the delivery of the intervention.

**Table 1:** Primary and secondary outcomes

Primary outcome	
The proportion of:	
● Neonatal deaths between enrollment and 72 h of age measured through vital status records every 12 h during hospital stay	
● Neonatal deaths between enrollment and 28 days of age measured through vital status records every 12 h during hospital stay and at a home visit on day 29 of age.	
Secondary outcomes	
The proportion of:	
● Infants receiving exclusive breastfeeding (or exclusive breast-milk feeding) at the end of the neonatal period measured by 24-h feeding recall at a home visit on day 29 of age. (Exclusive breastfeeding is defined as an infant receiving only breast milk and no other liquid or solid, with the exception of vitamin or mineral supplements, medicines, or oral rehydration solution, if prescribed.)	
● Infants with clinically suspected sepsis as per 12-hourly records during hospital stay.	
● Infants with hypothermia defined as any axillary temperature of less than 36 °C from 2 h after randomization until discharge (or 28 days of age if not discharged before then).	
● Infants with hypoglycemia defined as any blood glucose of less than 45 mg/dL (2.6 mmol/L) at mandatory measures at 6, 12, 18, and 24 h of age or at any other time.	
● Time to being fully breastfed: age at which the baby could feed fully by suckling on the breast without requiring any feeding by cup or nasogastric tube as per 12-hourly records.	
● Time to clinical stabilization: age at which the baby is considered to be clinically stable as per 12-hourly records and defined stability criteria.	
● Maternal satisfaction with health care in the hospital as per interviews.	
● Maternal depression defined as a score of 15 points or more in the Patient Health Questionnaire 9 (PHQ-9) administered to mothers at the day-29 home visit (Kroenke 2011).	

**Fig. 1:**
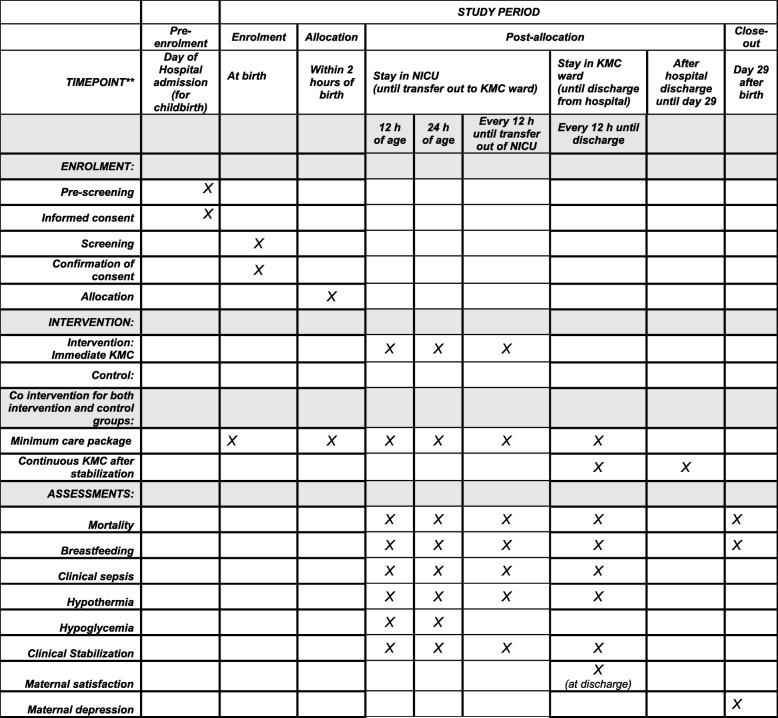
Schedule of enrollment, interventions, and assessments for the Immediate Kangaroo Mother Care (IKMC) Study

In addition, deaths from birth to 72 h of age of babies with a birth weight between 1.0 to less than 1.8 kg who are born in the participating hospitals but not enrolled in the study are reported. The schedule of outcome assessments is shown in Fig. 1.

### 2.8. Randomization

A computer-generated block randomization list with variable block size, stratified by site and by birth weight, has been prepared by a WHO statistician. The strata by birth weight are from 1.0 to less than 1.5 kg and from 1.5 to less than 1.8 kg. The random allocation is concealed in serially numbered, opaque, sealed envelopes prepared at the WHO and sent/delivered to the sites.

### 2.9. Blinding

The outcomes are assessed by an independent outcome measurement team which is not involved in the delivery of the intervention. However, the nature of the intervention prevents blinding of outcome assessors as they can observe whether the baby is with or without his or her mother in the NICU. Data analysts will be blinded to the allocation as far as possible.

### 2.10. Study implementation strategy

This clinical trial is conducted in compliance with the clinical trial protocol, good clinical practice, and the applicable regulatory requirements. The study is implemented in a standardized manner across all sites, and trial conduct is audited and monitored by quarterly visits by teams from the WHO. Fortnightly teleconferences track progress in the study and help to ensure ongoing harmonization. A full-time site trial coordinator at each site is responsible for the conduct of the trial. Three independent teams at each site are responsible for (i) screening and enrollment, (ii) KMC intervention support, and (iii) outcome measurement. Each team member has been trained in standard operating procedures relevant for their work.

### 2.11. The screening and enrollment team

#### 2.11.1. Pre-screening

An important ethical issue is that mothers will be asked to provide consent for participation when they are in a difficult situation (i.e., in the minutes and hours before and after birth). The nature of the intervention (starting KMC as soon as possible after birth) and the eligibility criteria (birth weight of 1.0 to less than 1.8 kg and no exclusion criteria) mean that the final confirmation of consent needs to be taken in the minutes after birth. The screening and enrollment team therefore “pre-screens” all pregnant women admitted for childbirth (including those who plan to have a caesarian section delivery) if they are not in advanced stages of labor and identifies pregnant women who are at high risk of delivering an LBW infant. Women with a gestational age of less than 37 weeks, intrauterine growth restriction based on second- or third-trimester ultrasound, age of less than 18 years, height of less than 1.50 m, multiple pregnancy, pre-eclampsia or eclampsia, severe anemia, or fundal height of less than 32 cm are identified as “high risk” for delivering an LBW infant. The research assistant approaches such pregnant women, informs them about the study, and invites them to participate in the study. Before taking consent, the treating physician or nurse/midwife is asked to certify that the woman is physically, psychologically, and emotionally fit to provide consent. Additionally, consenting mothers are asked to identify one or two adult women relatives or friends of their choice who could act as their surrogates for providing SSC when and if they are not able to do so. Surrogates have their role in the study explained to them if the mother–infant pair is randomly assigned to the intervention group.

#### 2.11.2. Screening

Health-care staff and a research assistant weigh every baby born in the hospital; the latter completes a screening form to assess whether the dyad meets all inclusion criteria and does not have any exclusion criteria. If mother and baby are eligible and the mother has consented prior to delivery, her consent is confirmed verbally before she and the infant are enrolled.

In situations where an infant is unexpectedly born very small (pre-screening either not carried out or if the pre-screening did not identify the pregnant woman as high risk), consent is obtained within the first 2 h of birth if the treating physician or nurse/midwife certifies that the woman is physically, psychologically, and emotionally fit. The mothers who consent after delivery are given another opportunity to decide about continued participation in the trial 24 h after birth. Mothers who are minors are eligible for enrollment in this study if they are at least 15 years of age and their consent is confirmed by the guardian (parent or husband). Women who undergo cesarean section and have not consented previously are not approached after delivery.

Recruitment of an adequate number of participants is ensured by pre-screening every pregnant woman admitted for child birth and screening every newborn born in the hospital. Inclusion of large public referral hospitals in the study and the proposed recruitment period of 2 years aim to ensure that the target sample size is adequately met. The WHO coordination team keeps a close watch on the progress of recruitment through two-weekly teleconferences.

#### 2.11.3. Enrollment and Randomization

The research assistant opens a sealed, opaque envelope with the study identification number, which has the group allocation inside, and records the assignment of the mother–infant pair to intervention or control groups. The research assistant informs the KMC support research assistant about the allocation.

### 2.12. KMC intervention support team

#### 2.12.1. Initiation of care according to group allocation

If the infant is allocated to the intervention group, the KMC support research assistant who attends all deliveries of potential babies helps the mother (or the surrogate in case the mother is indisposed) to initiate KMC as soon as possible after randomization. Monitoring of oxygen saturation and heart rate is carried out by using a pulse oximeter. The KMC support research assistant also helps to transfer the mother (or surrogate) and the baby to the NICU in SSC. The research assistant continues to support the mother or surrogate to provide continuous KMC after the baby is admitted to the NICU.

The infant is kept in SSC as much as possible, preferably with the mother but with a surrogate for the time when the mother cannot provide the intervention. In KMC, the infant is put naked on the mother’s chest. The infant has a cap, diaper, and socks and is secured firmly to the chest with a binder that ensures a patent airway and a shirt that provides containment in the fetal position. All routine care is provided in SSC. Any interruptions in SSC are documented to determine the duration for which the intervention was provided per day. The KMC research assistant also supports the mother in early expression of milk and to help the baby suckle at the breast.

If the infant is allocated to the control group, routine care is provided by hospital staff. The infant is transferred to the NICU as soon as possible. When discharged from the delivery room or operation theatre, the mother is transferred to the postnatal ward and the infant remains in the NICU in accordance with current guidelines. When the infant is ready to be fed, the mother provides expressed breast milk. When the infant is recovering, the mother provides brief sessions of KMC in the NICU in accordance with current WHO guidelines.

### 2.13. Outcome measure team

#### 2.13.1. Data collection

The research team assesses outcomes in the intervention and control groups by using identical methods and procedures. This independent team is not involved with the intervention delivery. Outcome measurement during hospital stay is through review of medical records (medical notes and treatment and feeding charts), interview with mothers, observation of the care given, and assessment of the baby, conducted every 12 h in the NICU and KMC ward. All forms completed by the outcome measurement, screening, and enrollment teams are entered in an electronic platform. Data range and consistency checks are incorporated into the data entry system. Facility-based data collection occurs up to hospital discharge. Several measures are in place to ensure participant retention and complete follow-up. When the mother–infant pair is ready for discharge from the hospital, field assistants accompany mother and baby home to get global positioning system (GPS) coordinates of the house, as well as to confirm contact information and obtain any alternative address where she would like to stay, to facilitate the last follow-up visit. This team builds and retains a strong rapport with the families to know their whereabouts during the follow-up periods. A home visit is conducted on day 29 of age to ascertain outcomes at the end of the neonatal period.

For those participants who withdraw from the study, the only data collected after withdrawal consist of in-hospital mortality.

Comprehensive monitoring of the safety of the study participants is performed throughout the course of the trial, from enrollment until the day-29 follow-up. Death of an enrolled baby is reported as a serious adverse event, and specific consideration is given for any factor potentially related to intervention. The investigator is responsible for informing the relevant local authorities, institutional review boards, or committees in accordance with their rules and standards. Once a report of an unexpected death is received, the WHO team informs the trial data and safety monitoring board (DSMB) within 24 h of receiving the information, and a final and detailed report is made within 1 week. The DSMB reviews these cases and makes recommendations regarding safety concerns and continuation of the study.

### 2.14. Monitoring of enrolled newborns

Every infant in the NICU is monitored, and information regarding temperature, heart rate, respiratory rate, and oxygen saturation is recorded every 6 h. Blood sugar similarly is monitored every 6 h during the first 24 h after birth and thereafter as and when required. In the KMC ward, infants are evaluated every 12 h up to hospital discharge. If signs of clinical deterioration are seen at any monitoring visit, the diagnosis of possible serious bacterial infection is considered.

### 2.15. Care for mothers

Unstable mothers or mothers who require special care are not transferred to the NICU. They continue to be managed in the intensive care unit or postnatal ward in accordance with usual hospital practice. The mothers are only transferred to the NICU for providing KMC once they no longer require special care.

Obstetric staff is responsible for providing postpartum monitoring and care for all mothers. If a mother is allocated to intervention, she receives routine postnatal care, including medical rounds, examinations, and medication, in the neonatal unit. A minimum care package for mothers is introduced for the postnatal care for mothers in both groups regardless of where they are located. The NICU nursing staff provides support in case of any emergency (such as secondary postpartum hemorrhage) and contacts the obstetric staff to provide definitive care.

### 2.16. Quality assurance

#### 2.16.1. Training and standardization

Health-care staff in the delivery room, neonatal unit, and KMC ward is trained to provide the neonatal care using the minimum care package for both the neonates and the mothers. All relevant staff of the neonatal unit and KMC ward as well as the KMC support research assistants are trained to support KMC for very small infants by the technical support team from Karolinska Institute. This includes how to secure them with the wrap to maintain the baby’s head in a safe position in order to keep the airway open, particularly when the baby is sleeping. All research assistants of the screening and enrollment, KMC support, and outcome measurement teams are also regularly trained in a standardized manual of operations for the study. All research staff is trained in rapport building and communication with mothers and families. Standardization exercises for assessment of clinical signs, including respiratory rate, temperature, chest indrawing, grunting, nasal flaring, and lethargy, have been carried out for all sites to ensure quality of study is maintained.

All research team members recruited for the study activities are well qualified and undergo intensive initial training. Their activities are supervised by competent trained study supervisors who support adherence to the manual of operations. The eligibility criteria and the outcomes to be assessed are very clearly defined in the manual of operations and the staff is trained and retrained regularly for that. The infant weighing scale is calibrated daily to minimize measurement errors. Internal quality checks are conducted by the trial coordinators and study principal investigators (PIs) at each site. The two types of quality checks are supervised observations and random independent checks. For the former, each research assistant is accompanied by the trial coordinator/PI for an activity each week. For the latter, 5% of observations are independently checked by the trial coordinator/PI.

All participating hospitals are supported to make quality-of-care improvements so they can implement the WHO Essential Care for Small Babies more effectively. The standard of care in accordance with the WHO manual for small infants includes monitoring, thermal control, breast-milk feeding support, and attention to hygiene for all infants. It also includes access to intravenous fluids, antibiotic therapy, and respiratory support with safe oxygen supplementation and bubble CPAP if required.

### 2.17. Trial registration

This trial is registered in two trial registries. The trial was first submitted for registration to Clinical Trials Registry - India (CTRI) in December 2017 as the India site was the first to be ready for implementation. It took a few months for clarifications and query resolution, and eventually the trial was retrospectively registered in CTRI (number: CTRI/2018/08/015369) on 17 August 2018. The trial was also registered in Australian New Zealand Clinical Trials Registry (ANZCTR) on 19 November 2018 once all of the African sites were enrolling in the trial (reference number: ACTRN12618001880235).

### 2.18. General principles for analysis

The primary analysis will be executed according to intention to treat. Even if KMC is discontinued because of clinical conditions of the mother or infant, the neonate will not be excluded. Effect size will be estimated with comparison of intervention and comparison group mortality risks. The two primary outcomes are complementary, so adjustment for type I error is not needed. Results will be reported by using Consolidated Standards of Reporting Trials (CONSORT) statement.

### 2.19. Flow of participants

**Fig. 2:**
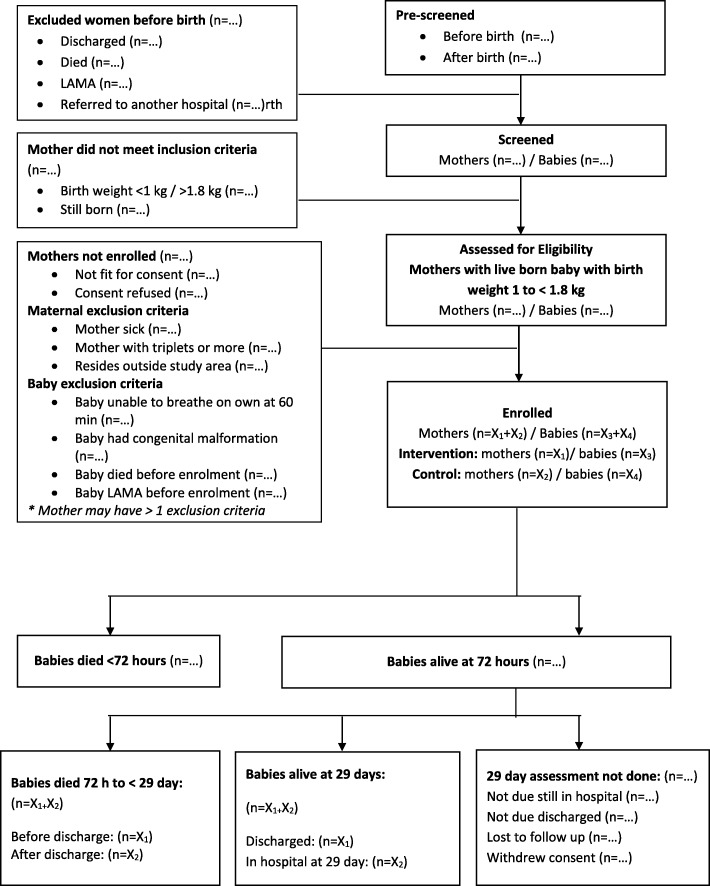
Structure of study flow chart

The flow and number of participants through assessment of eligibility, randomization, follow-up, and analysis are documented (Fig. 2). Reasons for exclusions and withdrawals are described.

### 2.20. Comparability of participants in the two groups

Descriptors for background characteristics in the intervention and control groups will be presented in a baseline table and summarized as means and standard deviations for continuous variables and as frequencies and percentages for categorical variables. These data will represent maternal age, parents’ schooling, family income, household characteristics, mode of delivery, birth weight, Apgar score, and age at randomization. Although our large sample size is likely to yield a balance between the two study arms in the main analyses, we will carefully evaluate the size of any baseline imbalance in our planned subgroup analyses. Imbalanced characteristics that predict death will be appropriately adjusted for statistically.

A second analysis comparing quality of care will be performed to ensure that the minimum package of care is offered equally to both intervention and control groups and that the intervention is delivered as planned. These will include time between birth to initiation of KMC and place of care in the delivery room or operating theatre until transfer to the NICU. Additionally, infants’ condition at the time of arrival to the NICU will be compared between groups.

### 2.21. Main effects

Effect sizes and their 95% CIs will be calculated for the primary outcomes. Significance tests with 5% significance level will be performed, and *P* values will be reported. If loss to follow-up for primary outcomes is below 2.5% for mortality, we will calculate RRs and their CIs. If loss to follow-up for primary outcomes is greater than 2.5%, hazard ratios and their CIs will be calculated. If important differences in baseline characteristics between intervention and control groups are identified, multiple logistic regression or Cox proportional hazards models will be used to adjust for confounding.

### 2.22. Subgroup analysis

Subgroup analysis will be conducted for the two mortality outcomes by (1) birth weight categories of 1.0 to less than 1.5 and 1.5 to less than 1.8 kg, (2) gestational age at birth categories of less than 34 weeks and 34 to less than 37 weeks, (3) singleton or twin births, (4) small for gestational age or not, and (5) mode of delivery (i.e., normal vaginal delivery or cesarean section). Statistical tests of interaction will be used to interpret whether the effect sizes in the categories are different or similar.

A secondary analysis stratified by compliance to immediate KMC (iKMC) over 72 h of age will be carried out. This analysis will report on efficacy of the intervention by average duration of SSC, classified as more than 20 h per day, 10–19 h per day, and less than 10 h per day. In this secondary analysis, reverse causality may be an important issue because severely ill newborns may receive less or no SSC. To reduce the possibility of reverse causality, in a sub-analysis we will exclude the babies who show signs of severe illness in the first 6 h of life.

### 2.23. Trial oversight

The trial steering committee is composed of all PIs from study sites, Karolinska Institute study consultants, and WHO technical staff functioning as its secretariat. This committee is responsible for designing and implementing the study in a harmonized way.

An independent DSMB has been established by the WHO. The DSMB includes seven members with expertise in clinical trials, statistics, newborn care, and ethics in resource-limited settings. The DSMB also serves as the technical advisory group for the trial. The DSMB is responsible for safeguarding the interests of trial participants, potential participants, investigators, and sponsors; assessing the safety and early efficacy of the trial’s intervention according to data available at a predefined schedule; monitoring the trial’s overall conduct and quality and protecting its validity and credibility; and making recommendations concerning continuation/termination of study determined by using O’Brien–Fleming stopping boundaries for early benefit/harm or futility. It also serves and advises the WHO and the PIs on the implementation of the trial. The members are independent of the trial and serve in their individual capacity. When about half of the enrollment is completed in the study, the DSMB will review an interim data analysis by arm to determine whether stopping boundaries have been crossed. The study protocol conforms to the SPIRIT checklist (Additional file 1).

## 3. Discussion

Upon completion, this trial will provide comprehensive data on safety and efficacy of iKMC in unstable LBW infants (birth weight of 1.0 kg to less than 1.8 kg) in developing countries. Furthermore, it will provide definitive answers on the impact of iKMC on neonatal mortality and on various outcomes in LBW infants, which will have implications for provision of clinical care. So far, KMC is recommended only for “stable” LBW infants. If the hypothesis is supported, this trial is likely to bring about a global paradigm shift in the intensive care of LBW infants.

Inclusion based on birth weight (as opposed to gestational age) is considered the best option to select the target population for three main reasons: (1) accurate gestational age estimation is unlikely in study settings where the ascertainment of the date of last menstrual period is generally not accurate and very few women have an ultrasound in early pregnancy, (2) there is high incidence of small-for-gestational-age infants who are also at a high risk of death and could benefit from SSC, and (3) it is relatively easy to have accurate measurement of birth weight.

Infants with a birth weight below 1.0 kg have reduced chances of survival even in high-resource settings with sophisticated neonatal intensive care. The intervention would be difficult to implement in infants with extremely LBW. Babies with birth weights of 1.8 kg or more are likely to be stable at or within the first hours after birth and therefore should not be separated from their mothers. They should be provided KMC based on current recommendation.

The iKMC trial is unique in several respects. First, it is a pragmatic trial conducted in the real-life scenario of public sector neonatal units in LMICs. The intervention is provided using existing clinical staff in these units, and research nurses are involved only in collection of the research data. Moreover, the trial uses exclusive clinical inclusion criteria and examines important clinical outcomes such as death, sepsis, and breastfeeding rates. The participating centers in the trial were carefully selected to ensure that representation from most of the LMICs and results would be applicable to the vast majority of public sector neonatal units in LMICs.

Second, a minimum level of quality of care for the neonates and their mothers is ensured in the trial by implementation and monitoring of the minimum care package in all study hospitals. This includes care and resuscitation at birth, thermal care, provision of CPAP or other adequate and safe respiratory support as needed, breastfeeding support, monitoring, prevention of infections, and management.

Third, the trial, while evaluating whether iKMC adds to the benefit of standard KMC for LBW survival, brings about a global paradigm shift of zero separation of mothers from their babies by introducing the concept of mother-neonatal intensive care unit (M-NICU). Continuous KMC for both groups is promoted and supported after they are transferred out of the M-NICU/NICU. This requires a higher level of collaboration between obstetric and neonatal departments.

If the trial demonstrates the safety and efficacy of iKMC in LMIC settings with reasonable intensive care facilities, the next logical step would be to scale up the intervention and implement M-NICUs across LMICs. The results from this trial would have implications for high income countries that routinely practice separation of mothers from their babies. Implementation of immediate KMC in these high income countries where newborn mortality is relatively low could improve the quality of non-mortality outcomes such as improved breastfeeding rates, maternal bonding, and long term child development outcomes.

### 3.1. Trial status

The trial is ongoing in all five sites: Ghana, India, Malawi, Nigeria, and Tanzania. The first participant was recruited on 1 December 2017. Participant recruitment is expected to be completed by September 2020. The current protocol is version 3.4, dated 29 March 2018.

## Supplementary information

**Additional file 1.** SPIRIT (Standard Protocol Items: Recommendations for Interventional Trials) 2013 Checklist.

## Data Availability

The datasets generated during the current study are available from the corresponding author on reasonable request.

## Abbreviations

CI: Confidence interval
CPAP: Continuous positive airway pressure
CTRI: Clinical Trials Registry - India
DSMB: Data safety monitoring board
iKMC: Immediate Kangaroo Mother Care
KMC: Kangaroo Mother Care
LBW: Low birth weight
LMIC: Low- to middle-income country
M-NICU: Mother-neonatal intensive care unit
NICU: Neonatal intensive care unit
PI: Principal investigator
RCT: Randomized controlled trial
RR: Risk ratio
SSC: Skin-to-skin contact
WHO: World Health Organization
